# Rethinking preeclampsia: six paradigm shifts in placental pathophysiology for maternal–foetal medicine

**DOI:** 10.3389/fmed.2026.1874122

**Published:** 2026-06-22

**Authors:** Can Bilginer, Ali Çetin

**Affiliations:** Department of Obstetrics and Gynecology, Haseki Training and Research Hospital, University of Health Sciences, Istanbul, Türkiye

**Keywords:** cellular senescence, extracellular vesicles, placenta, preeclampsia, syncytiotrophoblast, glycocalyx, angiogenic imbalance

## Abstract

Preeclampsia affects 2–4% of pregnancies worldwide and remains a leading contributor to maternal and perinatal morbidity and mortality. The prevailing framework, anchored in defective second-trimester spiral-artery remodelling, placental hypoxia, and antiangiogenic imbalance, continues to guide screening, prediction, and prevention. Evidence accumulated over the past 5 years, however, indicates that several pillars of this framework may require revision. This narrative review synthesises six inter-related paradigm shifts emerging principally from the research programme of the Huppertz group at the Medical University of Graz between 2020 and 2026, and situates them against contemporaneous mainstream formulations. The six shifts addressed are: first-trimester villous origins rather than second-trimester deep-placentation failure; intervillous hyperoxia rather than placental hypoxia in early-onset disease; metabolic and glycocalyx-based pathogenesis rather than pure angiogenic imbalance; the placenta as an endogenous exposome via extracellular vesicles; steroid imbalance coupled with alternative renin–angiotensin–leptin signalling; and reduced immune tolerance together with dynamic *in vitro* models that challenge inferences drawn from static explants. Collectively, these shifts reframe preeclampsia as a first-trimester syndrome of villous trophoblast dysregulation that propagates to maternal endothelial injury through multiple, partly redundant pathways. They suggest that maternal–foetal medicine may benefit from earlier, multimodal risk assessment, from biomarker panels that capture senescence, metabolic and extracellular vesicle signatures, and from a more cautious mechanistic interpretation of the soluble fms-like tyrosine kinase 1 to placental growth factor ratio, whilst acknowledging its established short-term clinical utility for triage. Several of these propositions, particularly those concerning intervillous hyperoxia and alternative renin–angiotensin–leptin signalling, still require independent replication in cohorts beyond the originating research environment before clinical translation. The six shifts do not individually overturn the two-stage framework; collectively they relocate the initiating lesion, broaden the signalling vocabulary, and argue for translation into first-trimester multimodal panels that extend beyond current angiogenic and Doppler measures.

## 1 Introduction

Preeclampsia complicates between 2 and 4% of pregnancies and accounts for approximately 46,000 maternal and 500,000 foetal or newborn deaths each year (1). Since the formulation of the two-stage placental model, mainstream pathophysiological thinking has held that insufficient transformation of the maternal spiral arteries during the late first and early second trimesters leads to chronic intermittent placental hypoxia, syncytiotrophoblast stress, and the release of antiangiogenic factors such as soluble fms-like tyrosine kinase 1 (sFlt-1) and soluble endoglin, which in turn generate maternal endothelial dysfunction (2, 3). This framework underpins the 2021 International Society for the Study of Hypertension in Pregnancy (ISSHP) recommendations (4), the Fetal Medicine Foundation competing-risks algorithm validated in the ASPRE trial (5), and the sFlt-1 to placental growth factor (PlGF) ratio-based rule-out strategy derived from the PROGNOSIS study (6) and extended through subsequent implementation trials and clinical guidance documents (7, 8).

Several observations, however, do not sit comfortably within this framework. Histological features of defective deep placentation are neither sensitive nor specific for preeclampsia and are also observed in uncomplicated pregnancies (9). Direct *in vivo* data on placental oxygenation have not consistently supported the inference of intervillous hypoxia, particularly in early-onset disease (10). Ratio-based biomarker tests are most informative within a narrow window close to clinical onset, implying that the antiangiogenic phenotype may reflect a relatively downstream step rather than the initiating lesion (7, 8). Low-dose aspirin initiated before 16 weeks of gestation reduces preterm preeclampsia by approximately 60% in the ASPRE cohort (5), a magnitude that is difficult to explain purely through second-trimester modulation of trophoblast invasion. A substantial minority of clinically definite preeclampsia arises in placentas without features of maternal vascular malperfusion, a finding that has motivated a revised two-stage model centred on syncytiotrophoblast stress rather than on invasion failure (3, 11).

Against this background, the research group led by Berthold Huppertz has advanced a family of concrete propositions that collectively amount to a reconceptualisation of preeclampsia (12, 13). The present review organises these propositions into six paradigm shifts, compares each with opposing views, and considers the implications for maternal–foetal medicine. The objective is to make the conceptual architecture visible to clinicians operating within the prevailing framework and to appraise the supporting evidence with appropriate epistemic caution.

### 1.1 Approach to literature selection

This work is a narrative, not a systematic, review. Primary literature was identified through structured searches of PubMed, Web of Science, and Scopus for English-language publications between January 2018 and March 2026. Search terms combined the descriptors “preeclampsia” or “pre-eclampsia” with “syncytiotrophoblast,” “glycocalyx,” “extracellular vesicles,” “senescence,” “first-trimester villous development,” “placental oxygenation,” “leptin,” “angiotensin,” and “flow culture.” Output of the Graz placental research programme led by Berthold Huppertz was retrieved with the author identifier and complemented by forward and backward citation tracking. The six paradigm shifts presented here were chosen on three criteria: (i) the proposition is articulated explicitly in one or more publications from the Graz programme between 2020 and 2026; (ii) the proposition addresses an element of the prevailing two-stage framework that recent independent evidence has rendered debatable; and (iii) the proposition has potential implications for first-trimester risk assessment, biomarker development, or clinical management. Topics for which no concrete clinical translation pathway could be identified were excluded. The synthesis prioritises mechanistic alignment over exhaustive coverage; the strength of evidence supporting each shift, including the extent of independent replication, is appraised in Table 1 and explicitly discussed in the corresponding sections.

**Table 1:** Comparative synthesis of the six paradigm shifts.

Paradigm shift	Traditional position	Proposed alternative	Strength of evidence	Independent corroboration	Clinical translation potential
1. First-trimester villous origins	Second-trimester spiral-artery remodelling failure is the initiating lesion (2, 14)	Villous trophoblast dysregulation and premature syncytiotrophoblast senescence precede invasion failure (12, 13, 16)	Developing	Independent senescence and oxidative-stress data (11, 17); cardiovascular model (9)	High; informs first-trimester multimodal panels
2. Intervillous hyperoxia in early-onset disease	Placental hypoxia drives oxidative stress and antiangiogenic release (20, 22)	Reduced transfer capacity produces intervillous hyperoxia with foetal hypoxia (10)	Emerging	Limited; classical oxidative-stress data partly compatible (20); awaits broader *in vivo* replication	Moderate; argues against maternal oxygen supplementation as a target
3. Metabolic and glycocalyx-based pathogenesis	Angiogenic imbalance is the central pathogenic mechanism (23)	Glycocalyx disruption and metabolic reprogramming lie upstream of angiogenic imbalance (24, 25)	Developing	Independent glycocalyx biomarker systematic review (26); HELLP–glycocalyx synthesis (27); soluble syndecan-1 cohort data	High; supports IPG-P, syndecan-1 and metabolic biomarker panels
4. Placenta as endogenous exposome via EVs	Soluble sFlt-1, sEng and cytokines mediate placenta–mother signalling (23)	Continuous EV flux delivers protein, lipid, microRNA and DNA cargo, partially bypassing soluble inhibitors (30)	Developing	Independent EV microRNA cohort (33); broader EV literature in obstetrics	High; first-trimester EV cargo panels under development
5. Steroid imbalance and alternative RAS–leptin axis	Endocrine and renin–angiotensin changes are secondary to placental dysfunction	Steroid precursor-to-product alterations and P-LAP-mediated leptin signalling are early bidirectional contributors (34, 35)	Emerging	Limited replication of P-LAP finding outside Graz; aligns with maternal cardiovascular model (9)	Moderate; potential first-trimester biochemical add-ons
6. Reduced immune tolerance with dynamic-model methodology	Immune maladaptation acknowledged as one of several contributors (19, 23)	CD24 reduction joins HLA-G downregulation and M1-shifted decidual macrophages; static explants understate secretory complexity (31, 36–38)	Developing	Independent HLA-G (37) and decidual macrophage polarisation (38) literature converges	Moderate; tolerogenic-marker panels worth prospective evaluation

Strength of evidence is graded as emerging when supported by one or two original studies, developing when supported by several converging studies including at least one outside the originating environment, and established when supported by multiple independent cohorts and incorporated into mainstream consensus statements. Clinical translation refers to first-trimester or near-term applicability for screening, prediction or management within 5 years. EV, extracellular vesicle; HLA-G, human leucocyte antigen G; IPG-P, inositol phosphoglycan P-type; PE, preeclampsia; PlGF, placental growth factor; P-LAP, placental leucine aminopeptidase; RAS, renin–angiotensin system; sEng, soluble endoglin; sFlt-1, soluble fms-like tyrosine kinase 1.

## 2 First-trimester villous origins rather than second-trimester deep-placentation failure

**Figure 1:**
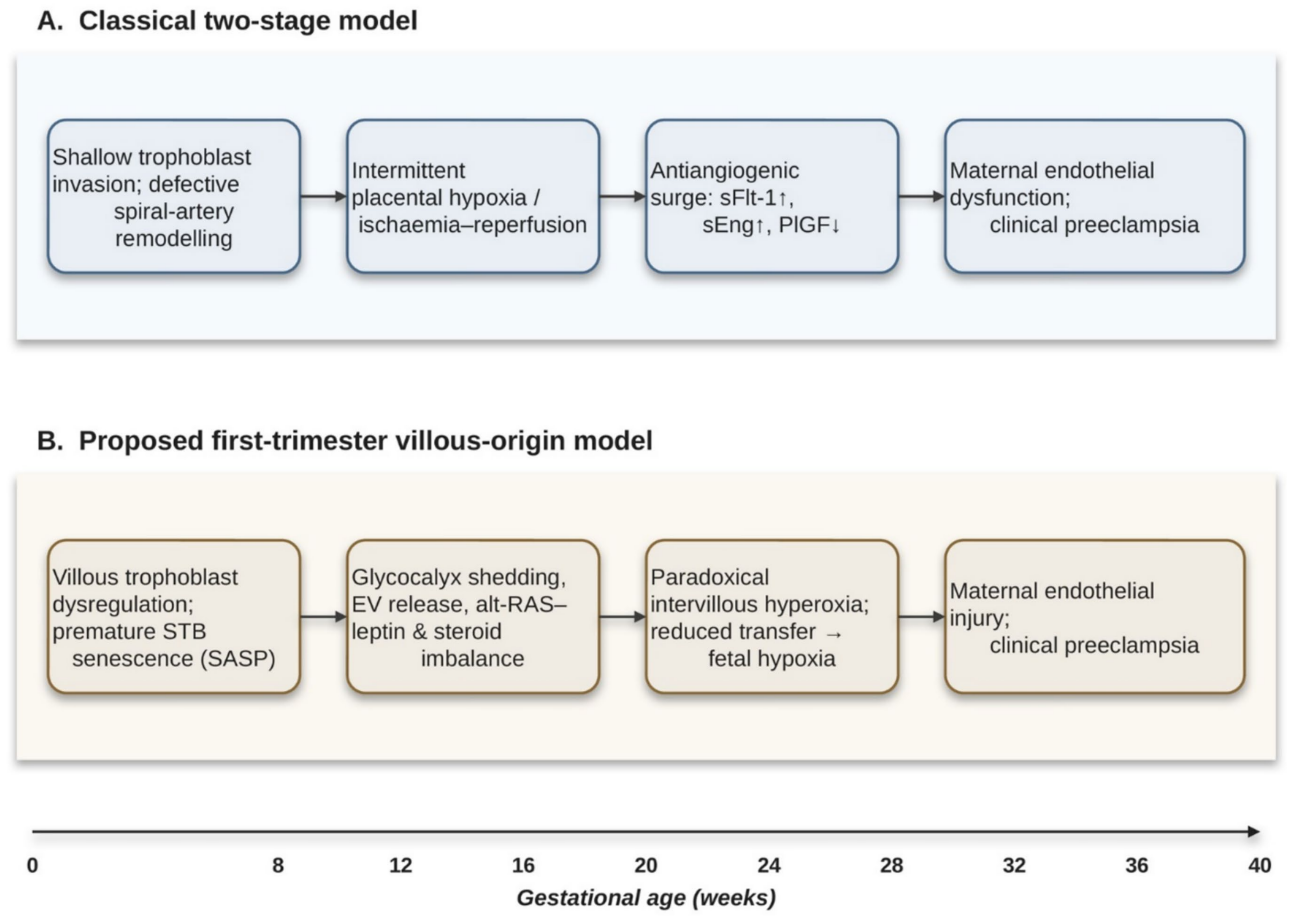
Temporal contrast between the classical two-stage model of preeclampsia **(A)** and the proposed first-trimester villous-origin model **(B)**. The classical sequence locates the initiating lesion in shallow trophoblast invasion at 8–18 weeks, with antiangiogenic imbalance emerging in mid-to-late gestation as a consequence of intermittent placental hypoxia. The proposed sequence relocates the initiating lesion to first-trimester villous trophoblast dysregulation, with premature syncytiotrophoblast senescence (SASP) driving glycocalyx shedding, extracellular vesicle release, alternative renin–angiotensin–leptin signalling and steroid imbalance, and culminating in paradoxical intervillous hyperoxia with reduced placental transfer capacity. In both models, the clinical syndrome of maternal endothelial dysfunction emerges in late gestation. The figure is intended as a conceptual schematic; the timing of individual events varies between published studies and between early- and late-onset phenotypes. STB, syncytiotrophoblast; SASP, senescence-associated secretory phenotype; EV, extracellular vesicle; alt-RAS, alternative renin–angiotensin system; sFlt-1, soluble fms-like tyrosine kinase 1; sEng, soluble endoglin; PlGF, placental growth factor.

The prevailing model locates the initiating lesion of preeclampsia in shallow interstitial and endovascular trophoblast invasion of the myometrial spiral arteries at 8 to 18 weeks of gestation (2, 14). In its revised form the model allows additional late-onset mechanisms related to placental size and senescence, but the early-onset phenotype remains anchored in deep-placentation failure (3, 11). Huppertz contends that this framing conflates an associated feature with the causal lesion. In the 2026 “Preeclampsia 2.0” series of opinion pieces, he argues that villous trophoblast dysregulation is detectable well before the classical invasion window and that defective spiral-artery remodelling is better interpreted as a consequence or parallel manifestation of upstream trophoblast dysfunction (12, 13). The argument is anchored in a detailed reconstruction of early human trophoblast lineage specification, in which syncytiotrophoblast formation, cytotrophoblast proliferation, and extravillous trophoblast differentiation are tightly coupled during the first trimester (15). The temporal contrast between the prevailing and the proposed sequence is summarised in Figure 1.

Mechanistic support has come from large-scale single-nucleus and spatial transcriptomic analysis of preeclamptic placentas, in which Nonn et al. identified dysregulated syncytiotrophoblast states consistent with a premature senescence-associated secretory phenotype, with several senescence-associated secretory factors detectable in maternal plasma as early as the first trimester (16). This observation aligns with wider placental senescence research that locates cellular ageing earlier and more diffusely than classical models anticipate (11) and is consistent with independent work linking oxidative-stress-induced senescence to early placental dysfunction in preeclampsia (17). The framework integrates with the demonstration by Cindrova-Davies et al. that human chorionic villi show no evidence of hypoxic stress during early placental development, even before full onset of maternal arterial perfusion (18).

Contrasting positions remain influential. Jung et al. maintain that uteroplacental ischaemia, mediated by failed spiral-artery transformation, is the principal upstream driver (19). Redman et al. and Sugulle et al. hold that both early- and late-onset phenotypes converge on syncytiotrophoblast stress, but that the origin of that stress in early disease continues to be located in defective placentation (3, 11). Melchiorre et al. invert the sequence and propose that maternal cardiovascular maladaptation precedes and produces placental dysfunction (9). The first-trimester villous hypothesis is compatible with the cardiovascular model, because dysregulated early villous trophoblast development could plausibly reflect, or respond to, constitutional maternal factors.

If the initiating lesion lies in first-trimester villous development, prediction strategies should move decisively upstream. The efficacy of aspirin initiated before 16 weeks (5) and the rise of senescence-associated secretory factors in first-trimester maternal plasma (16) both support this direction. Second-trimester uterine artery Doppler, although prognostically useful, may be better interpreted as a measure of a mid-cascade phenomenon rather than of the causal lesion.

## 3 Intervillous hyperoxia rather than placental hypoxia in early-onset preeclampsia

“Placental hypoxia” as a unifying explanation for the release of antiangiogenic factors has shaped preeclampsia research for two decades (20). In a systematic appraisal of published *in vivo* oxygenation measurements, Huppertz has argued that this concept is not consistently sustained by direct evidence (10). Across gestation, intervillous oxygen tension varies between roughly 20 and 60 mmHg; in early-onset preeclampsia with foetal growth restriction, the few available *in vivo* data sets indicate increased intervillous partial pressure of oxygen alongside reduced foetal partial pressure of oxygen (10). The interpretation offered is that diminished placental oxygen extraction, driven by reduced villous surface area and impaired transfer capacity, produces a paradoxical “hyperoxic placenta, hypoxic fetus” pattern rather than true maternal-side ischaemia. The number of *in vivo* studies remains small, and the interpretation should therefore be regarded as an emerging rather than an established position.

**Figure 2:**
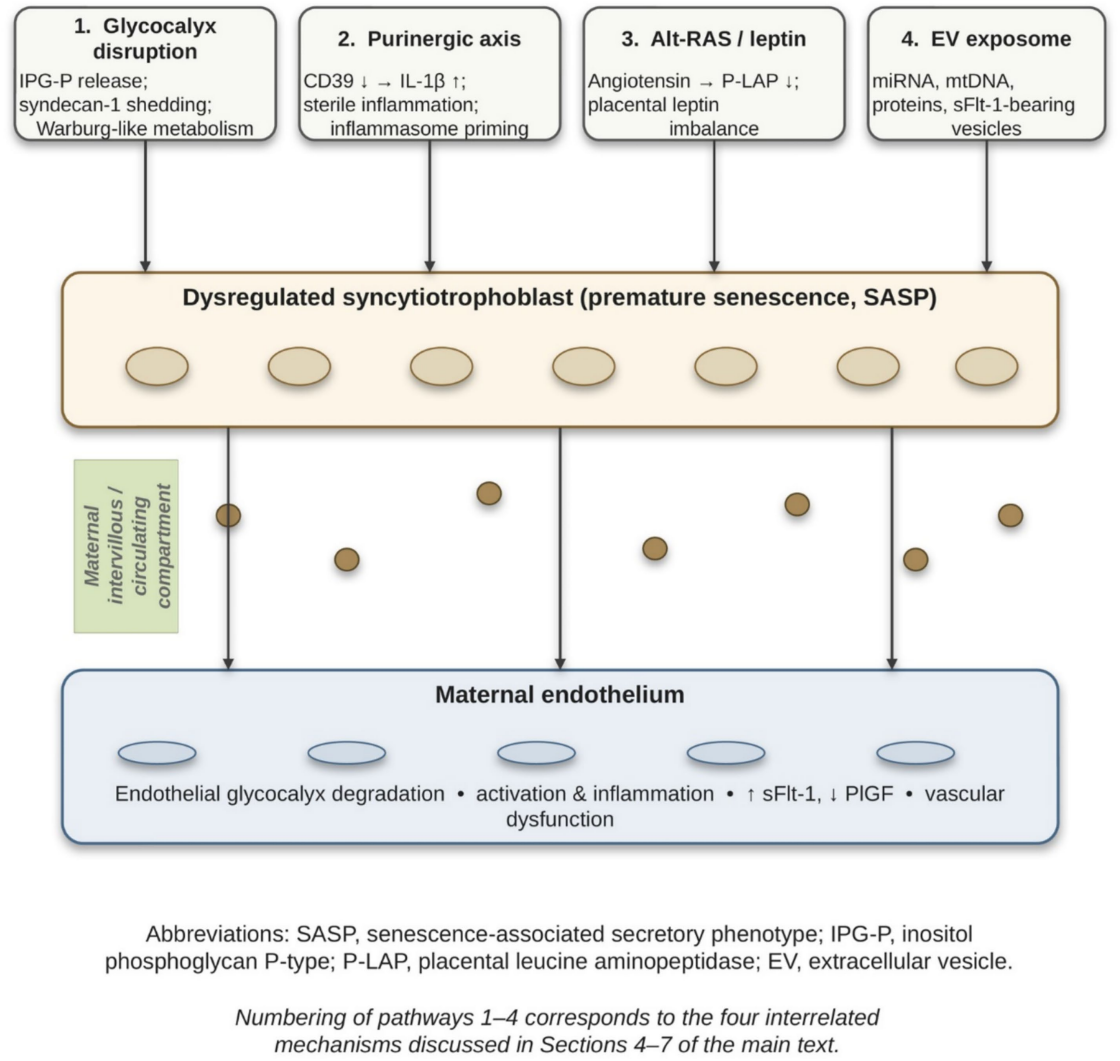
Schematic of the dysregulated syncytiotrophoblast in preeclampsia, integrating the four interrelated mechanistic pathways discussed in Sections 4 to 7. (1) Glycocalyx disruption releases inositol phosphoglycan-P mediators and syndecan-1 fragments and supports a Warburg-like placental metabolism. (2) Reduced placental CD39 expression removes a paracrine brake on platelet-derived factor-induced interleukin-1β production and primes sterile inflammation. (3) Lower placental leucine aminopeptidase shifts the angiotensin-driven control of placental leptin via the alternative renin–angiotensin–leptin axis. (4) Extracellular vesicle release exposes the maternal vasculature to microRNAs, mitochondrial and genomic DNA, surface ligands, and vesicle-bound sFlt-1, constituting an endogenous placental exposome. Convergence of these pathways on a premature senescence-associated secretory phenotype of the syncytiotrophoblast drives downstream maternal endothelial glycocalyx degradation, activation and inflammation, and ultimately vascular dysfunction. The diagram is conceptual; the temporal sequence of the four pathways is partially overlapping rather than strictly linear. SASP, senescence-associated secretory phenotype; IPG-P, inositol phosphoglycan P-type; P-LAP, placental leucine aminopeptidase; EV, extracellular vesicle; alt-RAS, alternative renin–angiotensin system; sFlt-1, soluble fms-like tyrosine kinase 1; PlGF, placental growth factor.

Two clarifications are warranted. The first concerns the mechanism by which a hyperoxic placental environment, in the absence of overt ischaemia, could nonetheless generate the syncytiotrophoblast stress and maternal endothelial injury that characterise preeclampsia. The proposed sequence runs from primary villous trophoblast dysregulation, through inadequate buffering of the physiological oxidative surge that accompanies onset of maternal arterial perfusion, to premature senescence of the syncytiotrophoblast (16, 17, 20). Under this account, sFlt-1 and soluble endoglin release, glycocalyx shedding, and extracellular vesicle vesiculation are downstream consequences of senescence-associated secretory activity rather than responses to ischaemia per se. The maternal endothelial injury that follows is therefore driven by a combination of antiangiogenic exposure and direct vesicle-mediated activation, not by hypoxia-induced placental ischaemia. The second clarification concerns terminology: “hyperoxia” denotes intervillous oxygen tension that exceeds the level expected for an actively transferring placenta, and is fully compatible with reduced transfer capacity and foetal hypoxia. The cellular and molecular elements of this sequence are summarised in Figure 2.

This reframing is consistent with the observation that oxidative rather than frankly hypoxic stress markers dominate in preeclamptic syncytiotrophoblast (17, 20) and with the classical demonstration that onset of maternal arterial perfusion generates a physiological oxidative surge that must be adequately buffered by trophoblast antioxidant systems (20). It also resonates with the finding that first-trimester chorionic villi do not display canonical hypoxia-inducible factor-driven hypoxic signalling (18). Automated artificial intelligence-based stereological methods, recently applied to the human placenta (21), offer the quantitative resolution required to test whether reduced villous surface density and capillary vascularisation underlie this transfer-capacity limitation.

Mainstream reviews nonetheless continue to present placental hypoxia as a central mechanism (19, 22). One reason is conceptual rather than empirical: most experimental models of preeclampsia, from the reduced uterine perfusion pressure rat model to *in vitro* hypoxia–reoxygenation paradigms, operationalise “placental stress” as oxygen deprivation (22). The counter-position is that these models capture consequences of reduced uterine arterial perfusion but do not necessarily recapitulate the oxygen environment inside the human intervillous space in early-onset preeclampsia (10). Independent *in vivo* data from groups outside the Graz programme remain limited, and the hyperoxia hypothesis should be regarded as biologically plausible but pending broader replication.

If the intervillous space is hyperoxic whilst foetal tissues are hypoxic, therapies aimed at improving maternal placental oxygenation, for example by maternal oxygen supplementation, may be misdirected. The framework supports a renewed focus on foetal oxygenation metrics, including middle cerebral artery Doppler and non-invasive assessment of foetal oxygen status, as complementary to maternal angiogenic profiles.

## 4 Metabolic and glycocalyx-based pathogenesis rather than pure angiogenic imbalance

The recognition that excess placental sFlt-1 and soluble endoglin disrupt maternal vascular endothelial growth factor and transforming growth factor-β signalling provided a mechanistic link between the placenta and the maternal syndrome and has dominated translational research (23). Several observations suggest that angiogenic imbalance may lie downstream of, rather than constitute, a primary metabolic and membrane-based perturbation.

Scioscia et al. have argued that disruption of the syncytiotrophoblast glycocalyx, a dense polysaccharide–proteoglycan layer, represents an earlier, upstream event (24). Glycocalyx disruption would simultaneously release inositol phosphoglycan-P mediators, which are linked to insulin-resistant, Warburg-like placental metabolism, expose membrane-anchored receptors to shedding, and facilitate vesiculation of sFlt-1- and soluble endoglin-rich microparticles. Under this account, the elevated sFlt-1 to PlGF ratio is interpreted as a marker of membrane turnover rather than a primary cause. The framework is consistent with a broader metabolic synthesis that highlights mitochondrial dysfunction and metabolic reprogramming as pivotal placental events in preeclampsia (25). Critically, this view has now been corroborated by independent groups outside the originating environment: a 2026 systematic review of circulating endothelial glycocalyx biomarkers in pregnancy concluded that syndecan-1, hyaluronic acid and heparan sulphate are robustly altered in hypertensive disorders of pregnancy (26), and a comprehensive 2025 narrative review proposed the endothelial glycocalyx as the missing link between angiogenic factors and systemic inflammation in preeclampsia and HELLP syndrome (27).

A second strand of work focuses on ectonucleotidase regulation. Forstner et al. showed that placental CD39 attenuates platelet-derived factor-induced interleukin-1β expression in human placental tissue, identifying a paracrine brake on sterile inflammation that appears to be compromised in preeclampsia (28). Together with evidence that decidual and tumour microenvironments share features of metabolic reprogramming and local immunosuppression (29), these data support a model in which dysregulated energy metabolism, purinergic signalling, and inflammasome priming operate upstream of the angiogenic imbalance that dominates clinical biomarkers.

The mainstream position, articulated by Rana et al. remains that imbalances in circulating angiogenic factors constitute the central pathogenic mechanism (23). This view is supported by the short-term rule-out performance of the sFlt-1 to PlGF ratio (6, 7), which retains substantial clinical utility for triage and short-term risk stratification in symptomatic women. These two positions are not incompatible: the membrane–metabolic account does not displace the angiogenic ratio from clinical use but offers a revised mechanistic interpretation in which the ratio reflects intermediate downstream events of placental membrane turnover. The clinical and mechanistic interpretations can therefore proceed in parallel.

If metabolic and glycocalyx-based pathology lies upstream, biomarker panels that include inositol phosphoglycan-P, glycocalyx shedding products such as syndecan-1, and purinergic metabolites may complement ratio-based algorithms. Metabolic modulators already under investigation for preeclampsia prevention gain additional mechanistic rationale from this framework.

## 5 The placenta as an endogenous exposome through extracellular vesicles

The traditional view of placenta-to-mother signalling emphasises soluble factors such as hormones, cytokines, sFlt-1, and soluble endoglin. Kupper and Huppertz have argued that this view is incomplete and have proposed the placenta as an “endogenous exposome” that exposes the maternal vasculature to a continuous, compositionally complex flux of extracellular vesicles carrying proteins, lipids, microRNAs, and in some fractions mitochondrial and genomic DNA (30). Unlike soluble factors, extracellular vesicles can deliver cargo selectively, activate endothelial and immune cells through surface-bound ligands, and partly bypass circulating inhibitors. This framework has been operationalised experimentally through the group’s placental flow culture system, which permits extracellular vesicle release to be studied under near-physiological mechanical conditions (31, 32).

Independent work has corroborated the extracellular vesicle-centred view. Aharon et al. reported distinctive maternal and placental extracellular vesicle microRNA signatures in gestational vascular complications, including preeclampsia, which distinguish placental from maternal contributions (33). Although sFlt-1 and soluble endoglin were historically conceptualised as purely soluble factors, they also circulate bound to or on extracellular vesicles, and the trajectory of vesicle concentration and cargo composition across pregnancy may offer a dynamic, patient-specific window into evolving disease that is less constrained than a single time-point ratio.

This paradigm does not replace but enlarges the angiogenic imbalance framework. Soluble and vesicle-bound antiangiogenic factors coexist in maternal plasma, and extracellular vesicles may act as a principal vehicle through which the placenta communicates danger signals during the earliest stages of the syndrome, with soluble factors predominating later.

Extracellular vesicle-based biomarkers open the prospect of detecting placental dysfunction in the first trimester using placental-specific surface markers combined with cargo profiling. Such panels may complement current combined screening and improve identification of women in whom aspirin prophylaxis is insufficient.

## 6 Steroid imbalance and the alternative renin–angiotensin–leptin axis

Steroidogenesis and the renin–angiotensin system have long been recognised as altered in preeclampsia, but these perturbations have generally been treated as secondary to placental dysfunction. Trummer et al. working within the Graz research environment, demonstrated that serum steroid profiles and precursor-to-product ratios in preeclamptic women exhibit specific alterations across multiple pathways spanning mineralocorticoid, glucocorticoid, and sex steroid metabolism, with changes detectable throughout gestation (34). These alterations are not trivially explained as epiphenomena of hypertension.

In parallel, Nonn et al. reported that maternal angiotensin signalling increases placental leptin expression in early gestation through an alternative renin–angiotensin system pathway operating via placental leucine aminopeptidase, the angiotensin IV receptor, rather than through the classical angiotensin II receptor type 1 axis (35). Lower placental leucine aminopeptidase expression was documented in preeclampsia and in first-trimester patients at high risk for preeclampsia, suggesting a mechanism that couples maternal cardiovascular regulation to placental endocrine output. This provides a bridge between maternal constitutional factors such as chronic hypertension, obesity, and insulin resistance, and early placental dysfunction. It also aligns with the maternal cardiovascular model advanced by Melchiorre et al. in which subclinical maternal cardiovascular maladaptation precedes and shapes placental development (9). Independent replication of the placental leucine aminopeptidase finding in non-Graz cohorts remains an outstanding requirement before the alternative renin–angiotensin–leptin axis can be incorporated into routine risk-stratification.

Taken together with the broader literature on altered maternal cortisol and aldosterone physiology in preeclampsia, these observations suggest that endocrine dyshomeostasis and alternative renin–angiotensin signalling are not solely late consequences but plausible early, bidirectional contributors.

Steroid precursor-to-product ratios and leptin trajectories measured in the first and early second trimesters may refine risk stratification beyond the current PlGF and sFlt-1 paradigm, particularly in women with pre-pregnancy cardiometabolic risk.

## 7 Reduced immune tolerance and dynamic models that challenge static assumptions

A sixth shift concerns both biology and methodology. Sammar et al. demonstrated that CD24, a glycosylphosphatidylinositol-anchored glycoprotein with documented immunoregulatory functions, is reduced in placentas from preterm preeclampsia (36). Because CD24 contributes to anti-inflammatory signalling at the maternal–foetal interface, its reduction implies diminished local immune tolerance and greater susceptibility to sterile inflammation driven by trophoblast debris and extracellular vesicles. This observation complements the CD39 and interleukin-1β data (28) and contributes to a coherent picture in which the preeclamptic placenta is both immunologically less restrained and more pro-inflammatory in its secretome.

The CD24 finding sits within a broader landscape of established immune-tolerance mechanisms at the maternal–foetal interface. HLA-G, the non-classical class I molecule expressed predominantly on extravillous and syncytiotrophoblast, restrains maternal natural killer-cell cytotoxicity and supports tolerogenic T-cell responses, and its expression and downstream signalling are reported to be downregulated in preeclamptic placentas alongside an upregulation of type-I interferon responses (37). Decidual macrophages, which under physiological conditions polarise predominantly towards the M2-like anti-inflammatory phenotype, shift towards an M1-like pro-inflammatory profile in preeclampsia, with implications for spiral-artery remodelling and trophoblast invasion (38). Decidual natural killer cells and regulatory T-cell function complete this network. CD24 reduction should therefore be read not as a stand-alone defect but as one component of a multilayered breakdown of tolerogenic signalling that includes HLA-G downregulation and a polarisation shift of decidual macrophages towards inflammatory phenotypes.

Methodologically, the group has argued that a persistent obstacle to progress has been the reliance on static placental explant culture, in which villous tissue is immersed in medium without physiological perfusion. Kupper et al. developed and validated a flow culture system that reproduces continuous intervillous perfusion ex vivo (31, 32). This system reveals that static and flow conditions yield quantitatively and qualitatively different secretomes, extracellular vesicle release profiles, and oxidative stress responses. Comparable insights come from systematic characterisation of trophoblast-derived cell lines, which documents substantial molecular divergence between immortalised and choriocarcinoma-derived lines and between any cell line and primary villous trophoblast (39). The revisited chicken chorioallantoic membrane assay further illustrates how tractable model systems can incorporate key features of maternal blood flow that are absent from conventional static explants (40). Independent spatial transcriptomic work (16) has likewise captured regional heterogeneity that static explants cannot resolve.

Mainstream statements on preeclampsia aetiology now acknowledge immune maladaptation as one of several contributory mechanisms (19, 23, 37), although the clinical translation of immune biomarkers has lagged behind angiogenic ones. CD24 and related tolerogenic markers, together with HLA-G expression patterns and decidual macrophage polarisation indices, merit evaluation as components of multimodal first- and second-trimester panels. More broadly, the recognition that much of the foundational mechanistic data in the field derives from non-perfused models should encourage caution when translating *in vitro* findings to clinical practise.

## 8 Clinical implications for maternal–foetal medicine

Taken together, the six paradigm shifts have concrete implications for how maternal–foetal medicine approaches preeclampsia. If preeclampsia originates in first-trimester villous trophoblast dysregulation, is associated with intervillous hyperoxia rather than hypoxia, involves metabolic and glycocalyx-based mechanisms alongside angiogenic imbalance, and communicates to the mother through extracellular vesicles and endocrine signals as well as soluble factors, the implications for screening, prediction, prevention, and management merit systematic reconsideration. The strength of evidence for each shift, and the corresponding clinical translation potential, are summarised in Table 1.

### 8.1 Screening

The ASPRE trial and subsequent work established that first-trimester combined screening using maternal characteristics, mean arterial pressure, uterine artery pulsatility index, PlGF, and pregnancy-associated plasma protein A (PAPP-A) detects approximately three-quarters of preterm preeclampsia at a 10% screen-positive rate (5, 41). The paradigm shifts argue for augmenting this model with markers of first-trimester villous dysfunction and early syncytiotrophoblast senescence (16), extracellular vesicle concentration and cargo (33), steroid precursor-to-product ratios (34), and glycocalyx shedding products (24, 26). Longitudinal analyses from the ASPRE cohort indicate that aspirin does not substantially alter PAPP-A or PlGF trajectories, implying that residual pathophysiology not addressed by the current intervention persists (42). Multimodal panels that incorporate these non-angiogenic dimensions may identify women in whom aspirin alone is insufficient.

### 8.2 Prediction

The sFlt-1 to PlGF ratio cut-off of 38 retains high short-term negative predictive value and remains useful for triage (6, 8); its established clinical utility is not disputed by the present synthesis. The mechanistic interpretation of the ratio may, however, be revised: in the glycocalyx–metabolic framework, the ratio reflects late membrane turnover, and elevated values warrant consideration of broader placental dysfunction in addition to a narrowly vascular interpretation. Serial PlGF-based testing, as evaluated in the PARROT trial, reduced the time to diagnosis of preeclampsia and was accompanied by a reduction in adverse maternal outcomes, establishing the clinical value of dynamic angiogenic biomarker measurement (43). Integrating such dynamic biomarkers with extracellular vesicle and steroid output trajectories (33, 34) may further refine individual risk estimation.

### 8.3 Prevention

Low-dose aspirin initiated before 16 weeks remains the best-validated intervention and reduces preterm preeclampsia by approximately 60% in high-risk women (5). The first-trimester origin hypothesis provides a mechanistic account of why early initiation matters: aspirin acts on the nascent villous trophoblast during its most plastic developmental window. Emerging preventive candidates, including pravastatin, metformin, and esomeprazole, share the property of modulating trophoblast metabolism, oxidative stress, or sFlt-1 release, and thus fit naturally within the metabolic–glycocalyx paradigm. The effects of maternal physical activity on placental structural adaptation observed in the DALI study suggest that lifestyle interventions may exert modest protective effects through related metabolic pathways (44).

### 8.4 Management and postpartum care

The recognition that preeclampsia reflects a broader placental–maternal exposome, with long-term cardiovascular consequences, supports structured postpartum surveillance and cardiovascular risk reduction, as advocated by the ISSHP (4, 45). Severe systemic inflammatory insults, as observed during the COVID-19 pandemic, can generate preeclampsia-like placental pathology through microthrombotic mechanisms, underscoring the view that multiple distinct upstream insults converge on a shared final pathway (3, 12, 46). These advances collectively support the expansion of biomarker discovery efforts beyond established placental biomarkers, with particular attention to multimodal panels that integrate angiogenic, metabolic, extracellular vesicle, and immune-tolerogenic dimensions.

## 9 Discussion

The six shifts outlined here do not constitute a single competing theory but a family of mutually reinforcing hypotheses that place the first-trimester villous trophoblast, its metabolism, its glycocalyx, its secretome, and its immune tolerance at the centre of preeclampsia pathophysiology. None of the shifts individually overturns the two-stage framework; collectively they relocate the initiating lesion, broaden the signalling vocabulary, and caution against over-reliance on static models and narrow biomarker panels.

The strength of the framework presented here lies in its capacity to reconcile several hitherto disparate observations: the magnitude of the ASPRE effect with aspirin initiated before 16 weeks, the narrow temporal window of utility for the sFlt-1:PlGF ratio, the heterogeneity of placental histopathology in clinically definite preeclampsia, and the persistent difficulty of translating *in vitro* mechanistic findings from static explants into clinically useful interventions. By relocating the initiating lesion to the first-trimester villous trophoblast, the framework aligns these observations with a coherent developmental sequence.

Important limitations and uncertainties remain. First, several of the mechanistic claims rest on studies with modest sample sizes from a single research environment. This is most marked for the intervillous hyperoxia hypothesis, where *in vivo* measurements are few and have not been replicated by independent cohorts; for the alternative renin–angiotensin–leptin axis, where placental leucine aminopeptidase data outside the Graz programme are limited; and for the steroid profile findings, which await prospective validation in geographically and clinically diverse populations. The glycocalyx and senescence components, by contrast, are supported by converging independent evidence (17, 26, 27, 37, 38) and are accordingly closer to clinical translation. Independent replication across geographically and clinically diverse cohorts is required before each of the proposed biomarkers can be integrated into routine practise. Second, the framework is synthesised retrospectively from a research programme whose hypotheses are still under active testing, and several specific propositions remain hypothesis-generating rather than confirmatory. Third, distinguishing cause from consequence in a developmental cascade that unfolds over the first half of gestation is methodologically demanding, and causal inference will require interventional or quasi-experimental designs that have yet to be implemented. Fourth, the present narrative review does not employ a systematic search protocol, and selection bias towards studies aligned with the proposed framework cannot be fully excluded.

Three priorities appear especially promising for translation. First, prospective validation of first-trimester multimodal panels incorporating senescence, extracellular vesicle, and steroid markers alongside established angiogenic and Doppler measures should be undertaken within adequately powered cohorts. Second, mechanistic dissection of glycocalyx and purinergic pathways as drug targets is required to move beyond the current dependence on aspirin as the sole evidence-based preventive intervention. Third, systematic comparison of static versus perfused placental models should be prioritised to clarify which conclusions in the existing literature require qualification. If these directions are pursued rigorously, preeclampsia may gradually cease to be the “disease of theories” and become a condition in which clinicians can match specific pathogenic trajectories to individualised early interventions.

## References

[1] Magee LA Nicolaides KH von Dadelszen P. Preeclampsia. N Engl J Med. (2022) 386:1817–32. doi: 10.1056/NEJMra2109523, 35544388

[2] Staff AC. The two-stage placental model of preeclampsia: an update. J Reprod Immunol. (2019) 134–135:1–10. doi: 10.1016/j.jri.2019.07.004 31301487

[3] Redman CWG Staff AC Roberts JM. Syncytiotrophoblast stress in preeclampsia: the convergence point for multiple pathways. Am J Obstet Gynecol. (2022) 226:S907–27. doi: 10.1016/j.ajog.2020.09.047, 33546842

[4] Magee LA Brown MA Hall DR Gupte S Hennessy A Karumanchi SA . The 2021 International Society for the Study of hypertension in pregnancy classification, diagnosis & management recommendations for international practice. Pregnancy Hypertens. (2022) 27:148–69. doi: 10.1016/j.preghy.2021.09.008, 35066406

[5] Rolnik DL Wright D Poon LC O’Gorman N Syngelaki A de Paco MC . Aspirin versus placebo in pregnancies at high risk for preterm preeclampsia. N Engl J Med. (2017) 377:613–22. doi: 10.1056/NEJMoa1704559, 28657417

[6] Zeisler H Llurba E Chantraine F Vatish M Staff AC Sennström M . Predictive value of the sFlt-1:PlGF ratio in women with suspected preeclampsia. N Engl J Med. (2016) 374:13–22. doi: 10.1056/NEJMoa1414838 26735990

[7] Dimitriadis E Rolnik DL Zhou W Estrada-Gutierrez G Koga K Francisco RPV . Pre-eclampsia. Nat Rev Dis Primers. (2023) 9:8. doi: 10.1038/s41572-023-00417-6 36797292

[8] Stepan H Galindo A Hund M Schlembach D Sillman J Surbek D . Clinical utility of sFlt-1 and PlGF in screening, prediction, diagnosis and monitoring of pre-eclampsia and fetal growth restriction. Ultrasound Obstet Gynecol. (2023) 61:168–80. doi: 10.1002/uog.26032, 35816445

[9] Melchiorre K Giorgione V Thilaganathan B. The placenta and preeclampsia: villain or victim? Am J Obstet Gynecol. (2022) 226:S954–62. doi: 10.1016/j.ajog.2020.10.024 33771361

[10] Huppertz B. Placental physioxia is based on spatial and temporal variations of placental oxygenation throughout pregnancy. J Reprod Immunol. (2023) 158:103985. doi: 10.1016/j.jri.2023.103985, 37406413

[11] Sugulle M Fiskå BS Jacobsen DP Fjeldstad HE Staff AC. Placental senescence and the two-stage model of preeclampsia. Am J Reprod Immunol. (2024) 92:e13904. doi: 10.1111/aji.13904 39049670

[12] Huppertz B. Preeclampsia 2.0: the urgent need for new thinking. J Reprod Immunol. (2026) 173:104820. doi: 10.1016/j.jri.2025.104820, 41353973

[13] Huppertz B. Preeclampsia 2.0: limitations and challenges of the two-stage hypothesis, and beyond. Mol Hum Reprod. (2026) 32:gaag001. doi: 10.1093/molehr/gaag001, 41495206 PMC12820888

[14] Erez O Romero R Jung E Chaemsaithong P Bosco M Suksai M . Preeclampsia and eclampsia: the conceptual evolution of a syndrome. Am J Obstet Gynecol. (2022) 226:S786–803. doi: 10.1016/j.ajog.2021.12.001, 35177220 PMC8941666

[15] Gauster M Moser G Wernitznig S Kupper N Huppertz B. Early human trophoblast development: from morphology to function. Cell Mol Life Sci. (2022) 79:345. doi: 10.1007/s00018-022-04377-0, 35661923 PMC9167809

[16] Nonn O Debnath O Valdes DS Sallinger K Secener AK Fischer C . Senescent syncytiotrophoblast secretion during early onset preeclampsia. Hypertension. (2025) 82:787–99. doi: 10.1161/HYPERTENSIONAHA.124.23362 39440423 PMC12002046

[17] Barbouti A Varvarousis DN Kanavaros P. The role of oxidative stress-induced senescence in the pathogenesis of preeclampsia. Antioxidants. (2025) 14:529. doi: 10.3390/antiox14050529, 40427411 PMC12108173

[18] Cindrova-Davies T Tissot van Patot M Gardner L Jauniaux E Burton GJ Charnock-Jones DS. Energy status and HIF signalling in chorionic villi show no evidence of hypoxic stress during human early placental development. Mol Hum Reprod. (2015) 21:296–308. doi: 10.1093/molehr/gau105, 25391298 PMC4339857

[19] Jung E Romero R Yeo L Gomez-Lopez N Chaemsaithong P Jaovisidha A . The etiology of preeclampsia. Am J Obstet Gynecol. (2022) 226:S844–66. doi: 10.1016/j.ajog.2021.11.1356, 35177222 PMC8988238

[20] Burton GJ Jauniaux E. Oxidative stress. Best Pract Res Clin Obstet Gynaecol. (2011) 25:287–99. doi: 10.1016/j.bpobgyn.2010.10.016, 21130690 PMC3101336

[21] Zafaranieh S Kummer D van Poppel MNM Desoye G Huppertz B. Automated stereological image analysis approach of the human placenta: surface areas and vascularization. Placenta. (2023) 142:115–8. doi: 10.1016/j.placenta.2023.09.002, 37688891

[22] Rana S Lemoine E Granger JP Karumanchi SA. Preeclampsia: pathophysiology, challenges, and perspectives. Circ Res. (2019) 124:1094–112. doi: 10.1161/CIRCRESAHA.118.313276 30920918

[23] Rana S Burke SD Karumanchi SA. Imbalances in circulating angiogenic factors in the pathophysiology of preeclampsia and related disorders. Am J Obstet Gynecol. (2022) 226:S1019–34. doi: 10.1016/j.ajog.2020.10.022, 33096092 PMC8884164

[24] Scioscia M Siwetz M Robillard PY Brizzi A Huppertz B. Placenta and maternal endothelium during preeclampsia: disruption of the glycocalyx explains increased inositol phosphoglycans and angiogenic factors in maternal blood. J Reprod Immunol. (2023) 160:104161. doi: 10.1016/j.jri.2023.104161, 37857160

[25] Manoharan MM Montes GC Acquarone M Swan KF Pridjian GC Nogueira Alencar AK . Metabolic theory of preeclampsia: implications for maternal cardiovascular health. Am J Physiol Heart Circ Physiol. (2024) 327:H582–97. doi: 10.1152/ajpheart.00170.2024 38968164 PMC11442029

[26] Fogacci F Yahya D Roeters Van Lennep J Borghi C Cicero AFG. Biomarkers of endothelial glycocalyx dysfunction in pregnancy: a systematic review of clinical relevance and detection techniques. Inflamm Res. (2026) 75:71. doi: 10.1007/s00011-026-02208-7, 41843118 PMC12995992

[27] Atallah A Sarda M-N McCarey C Massardier J Huissoud C. Endothelial glycocalyx: the missing link between angiogenic imbalance in preeclampsia and systemic inflammation in HELLP syndrome. Compr Physiol. (2025) 15:e70032. doi: 10.1002/cph4.70032, 40746214 PMC12314582

[28] Forstner D Guettler J Brugger BA Lyssy F Neuper L Daxboeck C . CD39 abrogates platelet-derived factors induced IL-1β expression in the human placenta. Front Cell Dev Biol. (2023) 11:1183793. doi: 10.3389/fcell.2023.1183793, 37325567 PMC10264854

[29] Krstic J Deutsch A Fuchs J Gauster M Gorsek Sparovec T Hiden U . (dis)similarities between the decidual and tumor microenvironment. Biomedicine. (2022) 10:1065. doi: 10.3390/biomedicines10051065 PMC9138511 35625802

[30] Kupper N Huppertz B. The endogenous exposome of the pregnant mother: placental extracellular vesicles and their effect on the maternal system. Mol Asp Med. (2022) 87:100955. doi: 10.1016/j.mam.2021.100955, 33612320

[31] Kupper N Pritz E Siwetz M Guettler J Huppertz B. Placental villous explant culture 2.0: flow culture allows studies closer to the *in vivo* situation. Int J Mol Sci. (2021) 22:7464. doi: 10.3390/ijms22147464, 34299084 PMC8308011

[32] Kupper N Pritz E Siwetz M Guettler J Huppertz B. *Ex vivo* placental explant flow culture: mimicking the dynamic conditions in utero. J Vis Exp. (2023) 199:e65919. doi: 10.3791/65919 37747189

[33] Aharon A Rebibo-Sabbah A Ahmad RS Dangot A Bar-Lev TH Brenner B . Associations of maternal and placental extracellular vesicle miRNA with preeclampsia. Front Cell Dev Biol. (2023) 11:1080419. doi: 10.3389/fcell.2023.1080419, 36910147 PMC9992195

[34] Trummer O Stern C Reintar S Mayer-Pickel K Cervar-Zivkovic M Dischinger U . Steroid profiles and precursor-to-product ratios are altered in pregnant women with preeclampsia. Int J Mol Sci. (2024) 25:12704. doi: 10.3390/ijms252312704, 39684415 PMC11640896

[35] Nonn O Fischer C Geisberger S El-Heliebi A Kroneis T Forstner D . Maternal angiotensin increases placental leptin in early gestation via an alternative renin-angiotensin system pathway: suggesting a link to preeclampsia. Hypertension. (2021) 77:1723–36. doi: 10.1161/HYPERTENSIONAHA.120.16425, 33775117

[36] Sammar M Siwetz M Meiri H Sharabi-Nov A Altevogt P Huppertz B. Reduced placental CD24 in preterm preeclampsia is an indicator for a failure of immune tolerance. Int J Mol Sci. (2021) 22:8045. doi: 10.3390/ijms22158045, 34360811 PMC8348750

[37] Boulanger H Bounan S Mahdhi A Drouin D Ahriz-Saksi S Guimiot F . Immunologic aspects of preeclampsia. Am J Obstet Gynecol Glob Rep. (2024) 4:100321. doi: 10.1016/j.xagr.2024.100321, 38586611 PMC10994979

[38] Wei Z Chen X Zhang J Lu X Huang X. Macrophage and preeclampsia: macrophage polarization imbalance at the maternal-fetal interface. J Clin Lab Anal. (2025) 39:e70046. doi: 10.1002/jcla.70046, 40343409 PMC12144585

[39] Pastuschek J Nonn O Gutiérrez-Samudio RN Murrieta-Coxca JM Müller J Sanft J . Molecular characteristics of established trophoblast-derived cell lines. Placenta. (2021) 108:122–33. doi: 10.1016/j.placenta.2021.02.022, 33810901

[40] Lyssy F Forstner D Brugger BA Ujčić K Guettler J Kupper N . The chicken chorioallantoic membrane assay revisited – a face-lifted approach for new perspectives in placenta research. Placenta. (2025) 166:77–84. doi: 10.1016/j.placenta.2024.04.013, 38705802

[41] Poon LC Shennan A Hyett JA Kapur A Hadar E Divakar H . The International Federation of Gynecology and Obstetrics (FIGO) initiative on pre-eclampsia: a pragmatic guide for first-trimester screening and prevention. Int J Gynaecol Obstet. (2019) 145:1–33. doi: 10.1002/ijgo.12802, 31111484 PMC6944283

[42] Rolnik DL Syngelaki A O’Gorman N Wright D Nicolaides KH Poon LC. Aspirin for evidence-based preeclampsia prevention trial: effects of aspirin on maternal serum pregnancy-associated plasma protein a and placental growth factor trajectories in pregnancy. Am J Obstet Gynecol. (2024) 231:342.e1–9. doi: 10.1016/j.ajog.2023.12.031, 38151219

[43] Duhig KE Myers J Seed PT Sparkes J Lowe J Hunter RM . Placental growth factor testing to assess women with suspected pre-eclampsia: a multicentre, pragmatic, stepped-wedge cluster-randomised controlled trial. Lancet. (2019) 393:1807–18. doi: 10.1016/S0140-6736(18)33212-4, 30948284 PMC6497988

[44] Zafaranieh S Siwetz M Leopold-Posch B Kummer D Huppertz B Desoye G . Placental structural adaptation to maternal physical activity and sedentary behavior: findings of the DALI lifestyle study. Hum Reprod. (2024) 39:1449–59. doi: 10.1093/humrep/deae090, 38733100 PMC11776022

[45] Melchiorre K Thilaganathan B Giorgione V Ridder A Memmo A Khalil A. Hypertensive disorders of pregnancy and future cardiovascular health. Front Cardiovasc Med. (2020) 7:59. doi: 10.3389/fcvm.2020.00059, 32351977 PMC7174679

[46] Garrido-Pontnou M Navarro A Camacho J Crispi F Alguacil-Guillén M Moreno-Baró A . Diffuse trophoblast damage is the hallmark of SARS-CoV-2-associated fetal demise. Mod Pathol. (2021) 34:1704–9. doi: 10.1038/s41379-021-00827-5, 34006935 PMC8130566

